# Roles and Transcriptional Responses of Inhibitory Neurons in Learning and Memory

**DOI:** 10.3389/fnmol.2021.689952

**Published:** 2021-06-15

**Authors:** Corinna Giorgi, Silvia Marinelli

**Affiliations:** ^1^CNR, Institute of Molecular Biology and Pathology, Rome, Italy; ^2^European Brain Research Institute (EBRI), Fondazione Rita Levi-Montalcini, Rome, Italy

**Keywords:** interneurons, memory, engram, IEGs, GABA, activity-dependent transcription, plasticity

## Abstract

Increasing evidence supports a model whereby memories are encoded by sparse ensembles of neurons called engrams, activated during memory encoding and reactivated upon recall. An engram consists of a network of cells that undergo long-lasting modifications of their transcriptional programs and connectivity. Ground-breaking advancements in this field have been made possible by the creative exploitation of the characteristic transcriptional responses of neurons to activity, allowing both engram labeling and manipulation. Nevertheless, numerous aspects of engram cell-type composition and function remain to be addressed. As recent transcriptomic studies have revealed, memory encoding induces persistent transcriptional and functional changes in a plethora of neuronal subtypes and non-neuronal cells, including glutamatergic excitatory neurons, GABAergic inhibitory neurons, and glia cells. Dissecting the contribution of these different cellular classes to memory engram formation and activity is quite a challenging yet essential endeavor. In this review, we focus on the role played by the GABAergic inhibitory component of the engram through two complementary lenses. On one hand, we report on available physiological evidence addressing the involvement of inhibitory neurons to different stages of memory formation, consolidation, storage and recall. On the other, we capitalize on a growing number of transcriptomic studies that profile the transcriptional response of inhibitory neurons to activity, revealing important clues on their potential involvement in learning and memory processes. The picture that emerges suggests that inhibitory neurons are an essential component of the engram, likely involved in engram allocation, in tuning engram excitation and in storing the memory trace.

## A Recap on Engrams

Higher cognitive functions such as learning, memory and processing of sensory perceptions, are strictly dependent on the correct flow of information within neuronal circuits made of both inhibitory and excitatory neurons. Further, it is the proper balance between excitation and inhibition that is particularly important for the correct execution of these brain functions.

Learning is the process during which an individual acquires a new information, thus representing the early phase of the formation or encoding of a memory. Memory, on the other hand, may be defined as the process that allows consolidation, storage and recall of the acquired information (Sherwood, 2015). The physical substrate of memory is assumed to be located in the so-called engram, a sparse ensemble of neurons that has the following properties: (i) is activated by a learning experience; (ii) has persistent structural and/or chemical modifications that affect their excitability and circuitry; (iii) is reactivated upon presentation of those same stimuli that triggered learning acquisition, resulting in memory retrieval (Reijmers et al., 2007; Liu et al., 2012; Josselyn et al., 2015; Josselyn and Tonegawa, 2020). Engram cells are thought to be connected into an “engram cell ensemble,” that once consolidated constitutes the cellular substrate of memory (Poo et al., 2016; Josselyn and Tonegawa, 2020). The current understanding is that neurons forming an engram ensemble are connected by strengthened synapses (Ryan et al., 2015), providing a conceptual link with the synaptic plasticity paradigm for learning and memory (Morris et al., 2003). This posits that the cellular correlate of memory-driven behavior is represented by the experimental phenomena of synaptic plasticity, characterized by enduring changes of synaptic strength such as long-term potentiation or depression (LTP or LTD) (Abraham et al., 2019). Accordingly, the encoding of information relies on the synaptic strengthening of both existing (Liao et al., 1995; Le Bé and Markram, 2006) and newly formed neuronal connections (Matsuzaki et al., 2004), along with increased excitability of ensemble neurons (Disterhoft et al., 1986; Oh and Disterhoft, 2015; Kim et al., 2016; Lisman et al., 2018).

How are active neurons allocated into an engram ensemble? According to Josselyn et al. (2015), during the encoding phase, active neurons in a given brain region compete against each other for allocation to an engram ensemble. Indeed, not all activated neurons will become engram cells supporting a particular memory: only a subset will be selected as engram ensemble neurons (memory allocation) based on their intrinsic excitability and increased synaptic strength (Zhou et al., 2009; Nonaka et al., 2014; Yiu et al., 2014; Gouty-Colomer et al., 2016; Park et al., 2016; Sehgal et al., 2018). Neurons with relatively increased intrinsic excitability, at the time of learning, win this competition to become engram cells (Han et al., 2007; Yiu et al., 2014). Several lines of evidence suggest that an additional mechanism shaping engram size involves inhibitory neurons (Holtmaat and Caroni, 2016; Morrison et al., 2016; Stefanelli et al., 2016; Josselyn and Tonegawa, 2020). Following excitatory plasticity, the potentiation of GABAergic activity may directly control the excitability of principal neurons by either disinhibition mechanisms (Donato et al., 2013; Cummings and Clem, 2020), or by lateral inhibition (Morrison et al., 2016; Stefanelli et al., 2016). While in the first, the enhancement of GABAergic neurotransmission causes an increase of projecting neurons excitability by inhibiting a relay interneuron, in the latter, the boost of inhibition triggered by activation of principal cells leads to inhibition of neighboring projecting neurons.

The size of an engram within a given brain region (that is, the number of engram cells in an ensemble) is stable and remains constant as memory strength increases (Han et al., 2007). Hence, memory strength does not affect engram size. Rather, an increased number and size of spines between engram cells underlies a stronger memory (Choi et al., 2018).

Once the engram ensemble is allocated, what makes it long-lasting? Increasing evidence suggests that memory persistence relies on structural and epigenetic modifications of engram neurons, which ultimately govern their connectivity and excitability (Frey et al., 1996; Kandel, 2001; Alberini and Kandel, 2014; Duke et al., 2017; Clayton et al., 2020). As described in a subsequent section of this review, these modifications are initiated at the very first stage of memory acquisition, whereby behavioral experiences trigger transcription and translation of activity-dependent genes. Expression of these immediate early genes (IEGs), including Arc, Fos, and Npas4, in turn activates diverse molecular programs which fundamentally modify synaptic strength, connectivity and epigenome of the activated ensemble. The persistence of such modifications is also dependent on activity-dependent expression of IEGs. Additionally, protein synthesis is crucial for the consolidation of a memory and it is via *de novo* translation of synaptic plasticity proteins that chemical and structural modifications (i.e., generation and deletion of dendritic spines, increase of synaptic strength and connectivity) become persistent (Bekinschtein et al., 2007; Matsuo et al., 2008; Yang et al., 2009; Sanders et al., 2012; Morris, 2013; Nonaka et al., 2014). Notably, with the exception of synaptic connectivity, these enduring changes are present in consolidated engram cells of a selected cellular memory trace (Ryan et al., 2015), but are lost in models of retrograde amnesia. For instance, in murine models of Alzheimer’s disease, or upon protein synthesis inhibition, engram neurons become “amnesic” as the increased spine density, enhanced synaptic strength and memory-guided behavior are lost. However, the connectivity between these neurons still persists and allows artificial memory retrieval (Ryan et al., 2015; Roy et al., 2016). Indeed, in amnesic animals, the natural memory recall is disrupted and only artificial (optogenetic) activation of “amnesic” but still connected engram cells, succeeds in memory retrieval. Therefore, while the long lasting potentiation of synapses is a prerequisite for encoding, formation and retrieval of memory traces (Garner et al., 2012; Ramirez et al., 2013; Nabavi et al., 2014; Lopez et al., 2015; Ryan et al., 2015), it may be dispensable for their storage once they are consolidated (Tonegawa et al., 2015).

The “engram paradigm” as discussed above, highlights neurons as the main component of the engram ensemble. Zooming in from this cellular level, to the finer spatial scale of the synapses, memory engrams can also be conceived as synaptic ensembles (Hayashi-Takagi et al., 2015; Poo et al., 2016; Gobbo et al., 2017), as indeed spines and synapses are considered the building blocks of memory (Roberts et al., 2010; Lai et al., 2012; Moczulska et al., 2013; Choi et al., 2018). Conversely, zooming out from the cellular level to the larger scale of neuronal networks, sets the focus on the interplay between inhibitory and excitatory neurons in engram ensembles.

In this regard, a major unresolved issue revolves around the cellular composition of engram ensembles and how different neuronal subtypes contribute to its formation and function. Current strategies adopted to map and manipulate memory engrams do not discriminate between the excitatory or inhibitory identity of the cells involved. Rather, independent evidence exists that engram includes both an inhibitory and an excitatory component. While principal neurons are often the target of memory trace studies, the inhibitory component of the engram is often overlooked. Nonetheless, increasing evidence shows that GABAergic inhibitory neurons play an integral role in engram formation, storage, and retrieval (Artinian and Lacaille, 2018; Lucas and Clem, 2018; Lamsa and Lau, 2019). Further, understanding how excitation and inhibition cooperate to form and maintain memory engrams is of pivotal importance. Hence, in this article, we review available evidence on the involvement of GABAergic interneurons in memory engram formation and function, with a focus on their emerging role in memory maintenance. In addition, we provide an overview of GABAergic interneuron transcriptional profiles in response to experience, extrapolating important hints on their contribution to learning and memory processes.

## Recruitment and Functions of Inhibitory Interneurons in Distinct Memory Phases

Over the last decade, a growing number of experimental and theoretical studies unveiled that the cooperation between GABAergic and glutamatergic neurons is fundamental to the shaping of different memory stages, including the encoding, consolidation, storage and recall (Vogels et al., 2011; Lucas and Clem, 2018). Hereafter, we will provide an overview of the role played by inhibitory neurons in distinct memory stages, detailing the mechanisms involved.

One route through which inhibitory neurons may be recruited to an engram is through signaling of neuromodulatory molecules. In an associative memory task, it has been shown that upon unconditioned aversive stimuli, acetylcholine elicits activation of somatostatin positive (SOM+) dendritic targeting-interneurons, promoting fear learning. This increase in GABAergic activity is required for memory formation, through inhibition of CA1 principal cells that received an enhanced excitation from the entorhinal cortex (Lovett-Barron et al., 2014). Another possible signaling that may contribute to GABAergic neuron recruitment into the engram ensemble is the dopaminergic one (Karunakaran et al., 2016). In this study, the authors found that activation of dopamine receptors is necessary to maintain high levels of parvalbumin positive (PV+) basket cell plasticity and ensures memory consolidation. Further, secretion of the brain derived neurotrophic factor (BDNF), elicited by Npas4 expression in pyramidal neurons of mice exposed to an enriched environment, increases the number of somatic inhibitory inputs onto principal cells, while their dendritic inhibition is decreased (Bloodgood et al., 2013). This redistribution of inhibitory circuitry may underlie fear memory formation, since the knockdown of Npas4 in the lateral amygdala, impaired memory fear encoding and retention (Ploski et al., 2011).

In addition to neuromodulatory molecules, homeostatic mechanisms may represent another potential route to engage GABAergic neurons in engram allocation. Indeed, both preclinical and clinical evidence revealed that an increase in inhibitory neuron activity is triggered in response to hyperexcitability of principal neurons and is required for proper brain functions (Barron et al., 2016; Fernandes and Carvalho, 2016; Chiu et al., 2019).

In the formation and consolidation of a memory trace, the potentiation of excitatory synapses among engram neurons, must be homeostatically compensated to prevent overexcitation of the neural network. Indeed, an increase in inhibitory synaptic plasticity can integrate excitatory plasticity in a memory network (Vogels et al., 2011). Stefanelli et al. (2016) observed that optogenetic stimulation of sparse granule cells induces an increased GABAergic response onto the dendrites of surrounding granule cells. This lateral inhibition of non-active principal cells relies on SOM+ interneurons activation and represents a mechanism to control engram size during formation of a memory trace. Likewise, in the lateral amygdala, a sustained activity of PV+ basket cells is necessary to shape engrams during the encoding of new information (Morrison et al., 2016). Overall, these studies point to a potentiation of GABAergic neurotransmission in controlling the engram size, during memory allocation.

GABAergic neurons can modulate activity of principal cells directly, or indirectly by targeting other interneurons (relay interneurons), which in turn contact excitatory neurons. This cellular arrangement, referred to as disinhibition, allows GABAergic cells to indirectly control the inhibitory tone onto glutamatergic neurons. Both direct inhibition and disinhibition play important roles in memory formation and recall. For instance, disinhibition is a common mechanism utilized to release the activity of excitatory engram cells in different phases of associative memory (Letzkus et al., 2015; Artinian and Lacaille, 2018).

During fear learning, exposure to an auditory cue induces excitation of PV+ interneurons which disinhibit amygdala principal cells by inhibiting SOM+ interneurons, with the final outcome of an enhanced auditory response and learning (Wolff et al., 2014). Another di-synaptic inhibitory circuit has been identified in the dorsolateral striatum of mice, in which PV+ interneurons control the output of medium spine neurons via neurogliaform interneurons, thereby improving learning (Lee et al., 2018). Subsequent independent studies observed an increased activity of cortical SOM+ neurons during both social fear expression and fear memory acquisition. This enhancement was necessary for both social fear and fear memory-guided behaviors. In particular, social fear and freezing behavior were dependent on SOM+ interneuron activation, which inhibits PV+ cells, ultimately disinhibiting cortical pyramidal neurons (Xu et al., 2019; Cummings and Clem, 2020). Thus, disynaptic disinhibition of pyramidal neurons, due to activation of upstream GABAergic cells, clearly contributes to memory encoding and recall. However, multiple studies converge in indicating that memory encoding is also accompanied by an increase in inhibitory drive directly onto activated pyramidal neurons. For instance, recent evidence finely identifies mechanisms regulating the perisomatic inhibitory plasticity onto activated CA1 pyramidal neurons (Yap et al., 2021). The authors observed that an enhanced and persistent perisomatic inhibition by PV+ interneurons onto Fos-activated pyramidal cells was counterbalanced by a weaker perisomatic inhibition by cholecystokinin expressing basket cells (CCKBC) (Yap et al., 2021). This bidirectional inhibition was regulated by Fos-induced Scg2 expression, whose suppression was shown to disrupt hippocampal network oscillations. The authors argue that Fos-driven reorganization of inhibitory inputs from PV+ and CCKBC may represent a mechanism for selecting a cell’s eligibility to take part in the encoding and recall phases of memory processes. Viceversa, activity-dependent expression of the IEG transcription factor NPAS4 in a subset of pyramidal neurons, selectively enhanced somatic inhibition mediated by CCKBCs, but not by PVBC (Hartzell et al., 2018).

Altogether, these studies emphasize the strategic functions of the inhibitory component of the engram in the early stages of memory formation.

A critical contribution of enhanced GABAergic activity was also observed in contextual fear memory consolidation. Indeed, pharmacogenetic inhibition of PV interneurons, in either CA1 or anterior cingulate cortex, disrupted brain oscillations along with memory strengthening (Ognjanovski et al., 2017; Xia et al., 2017). Additional evidence from CA3 and motor cortex points to a PV-network plasticity mechanism, induced by modulating VIP (vasoactive intestinal peptide)-PV microcircuits, as a requisite for long-term memory consolidation, retrieval and learning (Donato et al., 2013). In this study, motor learning tasks increased VIP+ inhibitory inputs onto PV+ cells. Upon completion of memory acquisition, excitatory inputs onto PV+ cells increased, leading to a network state dominated by inhibition. Hence, these studies highlight the potentiation of GABAergic activity as a process essential to memory maintenance.

Aside from the indirect di-synaptic disinhibition of pyramidal neurons, a straightforward reduction of inhibitory inputs, or of GABA levels, underlies the process of memory reinstatement after extinction-guided behavior and of memory recall (Courtin et al., 2014; Barron et al., 2016; Vallentin et al., 2016). During fear behavior, PV+ basket cell inhibition, achieved via optogenetic approaches or upon presentation of a neutral conditioned stimulus, directly increased the activity of cortical projecting neurons and reinstated the fear response after fear conditioning and extinction (Courtin et al., 2014). The inhibition of PV+ interneurons, as that of SOM+ cells, represents an important mechanism for gating fear learning. Aversive stimuli-induced interneuron inhibition led to an enhancement of the fear response by disinhibiting the entire somatodendritic compartment of principal cells, whose increased excitability is essential for memory trace acquisition (Wolff et al., 2014).

## The Inhibitory Component of the Engram in the Latent Phase of Memory Storage

As initially reported by Richard Semon in his description of the engram (Schacter, 2001), a consolidated memory trace lies in a dormant state before being reactivated by recall and memory retrieval.

So, what is keeping a memory latent in this storage phase? At the anatomical level, it is evident that reorganization of specific brain circuitry (i.e., hippocampus, cortex, amygdala) underlies memory storage in a time dependent manner (Bontempi et al., 1999; Frankland et al., 2004; Tonegawa et al., 2018). Nonetheless, the molecular mechanisms that support the retention of an engram are still unclear. The ongoing connections between engram cells, formed during learning and observed in silent engrams, may represent a way in which memory is retained (Tonegawa et al., 2015; Roy et al., 2017; Abraham et al., 2019). Unlike the normal and dormant engram cells, silent neuronal ensembles cannot be retrieved by natural stimuli and exhibit less increased synaptic strength and spine density (reviewed in Josselyn and Tonegawa, 2020). Additional mechanisms might be epigenetic modifications (Halder et al., 2016; Pearce et al., 2017; Perry et al., 2017; Clayton et al., 2020), wherein DNA methylation controls the maintenance of long-term memory changes in engram cells. Further, homeostatic plasticity processes (Turrigiano, 2012; Sprekeler, 2017; Goel and Dickman, 2021), such as synaptic scaling and potentiation of inhibition, could also contribute to memory retention. The potentiation of inhibition acts in response to an increased activity of postsynaptic projecting neurons to rebalance the hyperexcited neural circuitry (Marinelli et al., 2009; Lourenço et al., 2014; Chiu et al., 2018, 2019; Mendez et al., 2018). Modeling the activity pattern of spike rates, it emerged that the inhibitory connectivity, despite being equipped with a smaller number of synapses, determined these spike networks and was more effective in storing memory patterns than the excitatory connectivity. The rewiring of excitatory-to-excitatory connections did not have strong effects on the activity pattern of the spike network model, while the rewiring of the inhibitory-to-inhibitory connections led to a loss of memory recall. As a consequence, inhibitory circuits could have the potential to control the stability of a memory for a long time (Mongillo et al., 2018). Moreover, human studies revealed that an increase in cortical GABA concentration could represent a mechanism to keep memories latent. Indeed, a reduction in GABA levels, by anodal transcranial direct current stimulation, allowed a recall of dormant memories, suggesting that balanced excitatory-inhibitory neuronal ensembles are pivotal for the storage of memory traces in a latent state (Barron et al., 2016).

Earlier evidence indicated that during the initial stages of learning, pyramidal neurons of rabbit hippocampi showed increased spiking activity which decreased in later stages of learning, when the firing rate of theta cells (presumably interneurons) was enhanced (McEchron and Disterhoft, 1997). Similarly, a long-term enhancement of inhibitory synaptic transmission was recorded in mouse hippocampi, 5 days after an olfactory learning task (Ghosh et al., 2015). In keeping with these studies, anatomical structural plasticity evidence indicates an experience-dependent increase in feed-forward inhibition, paralleled by growth of inhibitory synapses onto CA3 pyramidal neurons for days after learning (Ruediger et al., 2011). This increased feedforward inhibition was required for memory precision and storage: photostimulation of PV+ interneurons promotes the enhancement of feedforward inhibition, maintaining the fear memory engram over time (Guo et al., 2018).

A recent paper underscores the importance of inhibitory synaptic plasticity occurring in parallel with excitatory synaptic plasticity, as a critical early step in preserving memory (Davenport et al., 2021). By optically controlling the translocation of alfa5-GABA_*A*_ receptors from extrasynaptic to synaptic inhibitory synapses, upon high-frequency stimulation of the excitatory input, a hidden and prolonged synaptic inhibition was revealed, that prevents accumulation of excitatory LTP. This form of metaplasticity, arising from the long-term changes of synaptic strength, may stabilize LTP/memory consolidation and may behave as an enduring event. Intriguingly, the inhibitory component of memory engrams may exert its functions through this form of plasticity.

All these lines of evidence suggest that potentiated inhibitory synapses onto principal neurons may be a necessary and sufficient condition for retaining engram cell ensembles in a dormant state until their reactivation upon memory recall.

The above reported studies prompt further investigations to elucidate if ensembles of active GABAergic neurons behave as the inhibitory component of engrams along the sequential phases of learning and memory. In particular, it remains to be experimentally ascertained whether GABAergic interneurons operate in memory processes as an integral component of the engram (e.g., allocated during learning, activated during storage and modulated during recall), or as accessory cells that merely gate the excitation of glutamatergic engram neurons.

Transcriptomic analyses are an emerging field of study that can significantly contribute to addressing these issues. As detailed in the following section, this approach has been utilized to interrogate the transcriptional response of specific neuronal subclasses to experience-induced activation or reactivation, unveiling new clues as to the contribution of inhibitory neurons to memory formation and recall.

## Activity-Dependent Transcription: a Closer Look at the Inhibitory Component of Memory Engrams

### Engram Mapping Strategies With Neuronal-Subtype Specificity

Critical advancements in memory engram mapping and manipulation have been enabled only recently by the ingenious combination of cutting-edge genetic tools exploiting the molecular cascades triggered by experience within engram neurons (Han et al., 2009; Liu et al., 2012). A common strategy adopted to label activated neuronal ensembles takes advantage of enhancer and promoter sequences of activity-dependent genes whose transcription is activated upon plasticity-inducing stimuli (Kawashima et al., 2014; DeNardo and Luo, 2017; Josselyn and Tonegawa, 2020). The promoters most widely utilized to aid neuronal activity mapping are those of the *F*os, Erg1, and Arc/Arg*3*.1 genes (Schilling et al., 1991; Waltereit et al., 2001; Kawashima et al., 2009; Pintchovski et al., 2009; Guenthner et al., 2013; DeNardo and Luo, 2017; Sauvage et al., 2019). Alternatively, the recently developed RAM system allows for active ensemble neuron labeling thanks to a synthetic promoter which combines the binding sites for the activity-dependent transcription factors Fos and Npas4 (Sørensen et al., 2016). As detailed below, however, expression of these well-known activity-dependent genes is not limited to one neuronal subtype, rather they appear to be induced in both excitatory and inhibitory neurons (Spiegel et al., 2014; Hrvatin et al., 2018). This implies that the engram labeling and manipulation strategies achieved, for example, through Fos-driven expression of optogenetic or chemiogenetic tools (DeNardo and Luo, 2017) are not restricted to a neuronal subtype. Rather, they perturb both GABAergic and glutamatergic components of the activated ensemble, yielding results that cannot be ascribed to either neuronal subtype.

Genetic strategies that can enable further restriction of engram manipulation to a specific neuronal subtype are now emerging, mainly combining subtype-specific CRE-driver mouse lines with a CRE-dependent expression of activity-driven effector genes. A promising example of this tactic is the CRAM construct, a CRE-dependent modification of the RAM system, which allows to investigate sub-populations of engram ensembles in a neuronal-subtype specific manner (Sørensen et al., 2016).

An alternative avenue to gain subtype specificity in engram manipulation could otherwise stem from the identification and exploitation of activity-dependent genes that are expressed only in a specific neuronal-subtype. While this characterization is still at its infancy, a growing number of studies investigating activity-dependent transcription in a cell-type specific manner, or at a single-cell level, are starting to emerge and represent an untapped resource. In the quest to investigate the functional contribution of GABAergic neurons to memory formation, consolidation and storage it is therefore important to take into account the activity-dependent transcriptional profiles of GABAergic neurons as a whole and of its numerous subclasses. Additionally, and as it will be discussed below, activity-dependent transcription critically contributes to the reorganization of synaptic connectivity within engram cells in response to activity. Thus, investigating the transcriptional responses of interneurons is a key step toward a deeper understanding of the mechanisms underlying the formation and functional responses of the inhibitory component of memory engrams. Hence, in the following sections, we will provide an overview of recent advancements in the field of cell-type specific activity-dependent transcription with a focus on GABAergic interneurons.

### Molecular Mechanisms Underlying Activity-Dependent Transcription

For many years, it has been broadly accepted that changes in gene expression patterns provide an essential contribution to the long-term modification of synaptic strength and to memory consolidation (Goelet et al., 1986; Nguyen et al., 1994; Alberini and Kandel, 2014; Minatohara et al., 2016). The current view is that, in response to experience, neuronal activity triggers both transcription of activity-dependent genes and chromatin modifications (Duke et al., 2017; Yap and Greenberg, 2018; Fernandez-Albert et al., 2019; Clayton et al., 2020), resulting in a substantially altered epigenomic and transcriptomic profile of that neuron. The initial transcriptional response is transient but leads to two major waves of activity-dependent gene expression with long-term effects. The first highly transient wave of transcriptional induction includes a set of genes, referred to as Immediate Early Genes (IEG), that encode several transcription factors (i.e., Fos, Npas4, Erg1-3) or a sub-set of plasticity-effector proteins (i.e., Arc, Amigo3) (Tyssowski et al., 2018; Yap and Greenberg, 2018). Sustained activity and these IEG transcription factors, in turn, elicit the second wave of late-response genes (LRG) expression. These include epigenetic modulators, secreted factors and neuromodulators (Mardinly et al., 2016; Su et al., 2017; Hrvatin et al., 2018; Jaeger et al., 2018), responsible for translating the transcriptional response into a functional one and promoting cell-type specific cellular and synaptic changes at the circuit level.

Importantly, while IEG transcriptional activation is independent from new protein synthesis, LRG activation is dependent on protein translation particularly of mRNAs encoding IEG transcription factors (Tyssowski et al., 2018). This distinction is important as it highlights the complex strategy utilized by neuronal cells to respond in a timely manner to activity without depending on the time-consuming protein translation process. Two molecular mechanisms have been described that contribute to ensure the fast kinetics of IEG transcription. Firstly, membrane depolarization triggered by neuronal activity induces calcium entry trough NMDA receptors and L-type calcium channels, initiating signaling cascades, primarily those involving the ERK/MAPK Kinases, which lead to the activation of pre-existing transcription factors such as the cyclic adenosine monophosphate (cAMP)-responsive element binding protein (CREB), the myocyte enhancer factor 2 (MEF2), and serum response factor (SRF) (Kawashima et al., 2009; Pulimood et al., 2017; Tyssowski et al., 2018). These transcription factors, in turn, activate transcription of almost all known IEGs. A second mechanism contributing to a rapid induction of IEG genes relies on their promoters being predisposed to swift activation by recruitment of Poised PolII complexes and open chromatin states (Saha et al., 2011; Tyssowski et al., 2018).

The molecular cascades underlying activity-dependent transcription are common to most neuronal cells analyzed. However, the resulting pool of mRNAs induced in different areas of the brain and in different cell types is strikingly specific. Upon learning, distinct brain regions display highly divergent transcriptional profiles, as best exemplified in recent transcriptomic analyses performed from dorsal hippocampi and prelimbic cortices of mice subjected to inhibitory avoidance learning (Katzman et al., 2021). As detailed below, great heterogeneity also characterizes the activity-dependent transcriptional profiles of different neuronal subtypes in the same brain region, reflecting and facilitating their functional specialization.

### Subtype Specificity of Activity-Dependent Transcription

Activity-dependent gene expression has been investigated for decades; however, early studies have been limited in resolution and detection sensibility as they were based on pharmacologically induced generalized activation applied on cultured neurons or bulk tissues (Loebrich and Nedivi, 2009).

**FIGURE 1:**
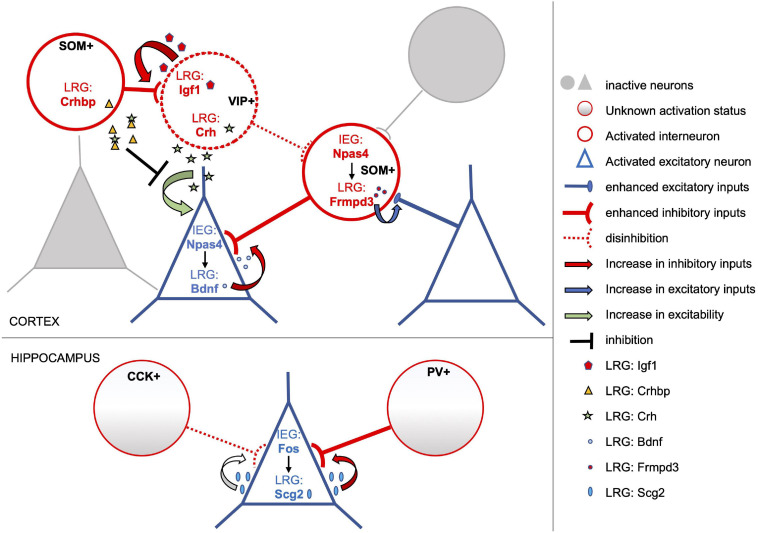
Subtype-specific activity-dependent transcriptional programs shape circuit rearrangements in response to experience. The model depicts examples of activity-induced LRGs which modulate connectivity and/or excitability of distinct neuronal subtypes, overall enhancing the inhibitory tone onto activated pyramidal neurons. Excitatory and inhibitory neurons of the activated neuronal ensemble are depicted as blue triangles and red circles, respectively. In cortical excitatory neurons the IEG Npas4 drives expression of several LRGs including Bdnf (blue dots), which in turn promote an increase in the number of inhibitory inputs onto these cells. Conversely, Npas4 expression in activated SOM + inhibitory neurons triggers a transcriptional program that includes the LRG Frmpd3 (red dots), which induces an increase in excitation onto the GABAergic cell (Spiegel et al., 2014). Activity-dependent transcriptional profiles of cortical VIP + interneurons include the LRGs Igf1 (red pentagons) and Crh (green stars). Secreted Igf1 promotes inhibition of VIP + neurons (Mardinly et al., 2016), which often operate by disinhibiting principal neurons. On the other hand, VIP + neurons also release Crh which can increase the excitability of nearby pyramidal cells. However, this effect may be tampered by Crhbp (yellow triangles), an inhibitor of Chr secreted by SOM + neurons in response to stimulation (Hrvatin et al., 2018). Overall, activity-dependent transcriptional responses of both excitatory and inhibitory cortical neurons appear to converge in increasing the inhibitory tone onto activated excitatory neurons. Similarly, in the hippocampus, Fos expression in activated excitatory neurons induces the LRG Scg2, a precursor to four neuropeptides, which increases perisomatic inhibition by PV-interneurons albeit weakening that of CCK-interneurons (Yap et al., 2021).

The first study to pioneer a comprehensive analysis of neuronal subtype specificity in activity-driven transcriptomes was carried out by Spiegel et al. (2014), comparing the transcriptional response of cortical inhibitory neurons to that of excitatory neurons both *in vitro* and *in vivo*. Microarray analyses revealed that, at 1 h of KCl depolarization, cultures enriched of excitatory neurons or of GABAergic neurons express a highly overlapping set of IEGs. In particular, all but one of the transcriptional regulators acutely induced by depolarization in GABAergic cultures were found also induced in excitatory neurons. These common IEGs include Fos, FosB, Egr1-3, Nr4a1, and Npas4. Strikingly, the well-studied plasticity-effector protein Arc, commonly considered a primarily glutamatergic IEG (Vazdarjanova et al., 2006; Morrison et al., 2016; Nikolaienko et al., 2018), resulted among the IEG induced in both cortical interneurons and excitatory neurons in culture. However, substantial differences in the transcriptional profiles of inhibitory and excitatory neurons clearly emerge when the authors investigate the second wave of LRG induction. At 6 h from depolarization, they found only a minimal overlap (25% of LRG transcripts) in the transcriptional responses of inhibitory and excitatory neuronal cultures. With this approach, for the first time, hundreds of late response genes specifically induced in activated interneurons have been identified, including Cacng5, Igf1, Pnoc, Pthlh and E530001K10RIK (Table 1). Surprisingly, this analysis also revealed that several well-known LRGs, such as Bdnf, Homer1 and Cpg15/Nrn1, are selectively induced in excitatory neurons and not in inhibitory neurons. These important findings were also corroborated *in vivo*, through identification of ribosome-associated transcripts within cortical GABAergic or glutamatergic neurons in Ribo-Tag mice (Sanz et al., 2009) undergoing visual stimulation. Hence, in this study, both *in vitro* depolarization and *in vivo* visual stimulation induced a similar set of immediate early genes in inhibitory and excitatory neurons. However, depending on the neuronal subtype, this same set of transcription factors appears to drive distinct downstream LRG transcriptional profiles, possibly as a consequence of differential epigenetic modifications and accessibility of their target enhancer and promoter elements in each neuronal subtype (Yap and Greenberg, 2018). To further investigate this important finding, Spiegel et al. (2014) proceeded to assess the role of the IEG transcription factor Npas4 in driving LRG transcriptional profiles in excitatory vs. inhibitory neurons. Npas4 had been previously suggested to regulate the inhibitory-excitatory balance of neural circuits and was shown to promote perisomatic inhibition of excitatory neurons (Lin et al., 2008; Coutellier et al., 2012; Bloodgood et al., 2013). Notably, the increase of inhibitory synapses onto the soma of pyramidal neurons elicited by Npas4 requires Bdnf, whose induction is restricted to excitatory neurons. Thus, Npas4 represents an ideal example of a shared IEG transcription factor that may induce distinct transcriptional responses in distinct neuronal subtypes. Depleting its expression in SOM+ interneurons specifically, the authors (Spiegel et al., 2014) find that Npas4 does not affect the number of inhibitory synapses formed onto these cells, in stark contrast with its role in excitatory neurons. To the contrary, Npas4 expression is required to promote formation of excitatory synapses onto SOM+ neurons in response to activation, likely by eliciting expression of LRGs which participate in modifying excitatory synaptic inputs. Indeed, a characterization of Npas4-regulated LRGs in excitatory and inhibitory neurons, confirms that this transcription factor induces distinct transcriptional programs in the two neuronal subtypes. In particular, four LRGs (Frmpd3, scl25a36, Kcna1, Ddhd1) were identified as specifically induced in SOM+ neurons activated *in vivo*, and several of these have been suggested to be involved in excitatory synapse formation and stabilization. Overall, these results suggest that in excitatory and inhibitory neurons activity triggers a common set of IEG which, in turn, elicit the expression of different combinations of LRGs resulting in subtype-specific synaptic responses to activity (Table 1). This mechanism is well exemplified by the role played by the IEG Npas4 in the homeostatic response to neuronal activation (Figure 1); while in excitatory neurons it elicits an increase in Bdnf expression and a recruitment of inhibitory inputs, in SOM+ GABAergic neurons it induces a different LRG transcriptional response resulting in an increase of excitatory inputs onto these cells. Interestingly, a follow-up study by the Greenberg laboratory revealed that, in the visual cortex, the transcriptional and functional response to activation of various interneuronal subclasses differ substantially (Mardinly et al., 2016). Using the Ribo-Tag approach, the authors profiled actively translated mRNAs at different time points from visual stimulation in SOM+, VIP+, and PV+ interneurons, yielding 31 genes that are both regulated by sensory experience and specific to a neuronal subtype. Remarkably, most of these cell-type specific activity-dependent genes encode proteins that are secreted and could thus play a role in modulating the synaptic connectivity of the activated interneuron. For example, of the 11 experience-regulated genes specific to VIP+ neurons, four code for secreted molecules and are Igf1, Crh, Prok2, and Fbln2. FISH analyses then revealed that Fbln2 is expressed at undetectable levels, while Prok2 is sparsely expressed only by a subset of VIP+ neurons upon stimulation of the visual cortex. Crh was instead induced in all VIP+ neurons, but also in other SOM+ /PV+ /VIP-negative interneurons. The authors thus proceed to investigate the function of Igf1, which is specifically enriched in most stimulated VIP+ neurons. Its selective depletion and overexpression in these cells revealed that Igf-1 acts locally to promote the number and/or strength of inhibitory synapses formed onto VIP+ neurons in response to activity and does so in a cell autonomous manner (Figure 1). Further, Mardinly et al. (2016) show that experience-dependent induction of Igf-1 negatively regulates visual acuity. Since VIP+ neurons are involved in disinhibition (Pi et al., 2013) of cortical circuits, this study indicates that IGF-1 is secreted locally by VIP+ neurons to enhance their own inhibition, thereby putting a brake on the excitability of targeted principal neurons in response to experience.

**TABLE 1:** Examples of IEGs and LRGs induced in inhibitory neurons.

TABLE 1A						
**IEG name**	**Brain area**	**IN subtype**	**Activity-dependent induction in other cell types**	**Induction protocol**	**Method**	**Ref.**

Egrl	Vis. CX	Gad2+, VIP+, SOM+, PV+	yes (EX_Emxl+)	visual stim.	Ribo Tag/Gad2-CRE	1,2
	Vis. CX	all IN, high in SOM+	yes (EX, OPC, PE)	visual stim.	scRNA-seq	5
	me A	CCK+, SOM+	yes (EX, AS, MG, OPC, EC)	seizure (PTZ)	Act-seq	3
Erg4	me A	Gad1+, Gad2+	yes (EX, AS)	seizure (PTZ)	Act-seq	3
	Vis. CX	SOM+	yes (EX)	visual stim.	scRNA-seq	5
Fos	Vis. CX	Gad2+, VIP+, SOM+, PV+	yes (EX_Emxl+)	visual stim.	Ribo Tag/Gad2-CRE	1,2
	Vis. CX	all IN	yes (EX, EC, SM, MA, AS)	visual stim.	scRNA-seq	5
	meA	Gad1+, Gad2+	yes (EX, AS, OPC, EC, MC)	seizure (PTZ)	Act-seq	3
FosB	Vis. CX	Gad2+, VIP+, SOM+, PV+	yes (EX_Emxl+)	visual stim.	Ribo Tag/Gad2-CRE	1,2
	Vis. CX	SOM+, VIP+	yes (EX)	visual stim.	scRNA-seq	5
	meA	Gad1+, Gad2+	yes (EX, EC, MC)	seizure (PTZ)	Act-seq	3
Npas4	Vis. CX	Gad2+, VIP+, SOM+, PV+	yes (EX_Emxl+)	visual stim.	Ribo Tag/Gad2-CRE	1,2
	Vis. CX	VIP+	yes (EX)	visual stim.	scRNA-seq	5
JunB	meA	CCK+	yes (EX, AS, OPC)	seizure (PTZ)	Act-seq	3
	Vis. CX	PV+, SOM+, VIP+	yes (EX)	visual stim.	scRNA-seq	5
Nr4a3	cortex	Ndnf+, Vip+	yes (EX,EC,MG AS, OPC)	seizure (PTZ)	snRNA-Seq	4
	Vis. CX	SOM+, VIP+, Npy+	yes (EX,SM,MA,AS)	visual stim.	scRNA-seq	5
	meA	Gad1+, Gad2+	yes (EX, OPC)	seizure (PTZ)	Act-seq	3
Fosl2	Vis. CX	all IN	yes (EX,EC,SM,MA,AS,PE)	visual stim.	scRNA-seq	5
Tiparp	cortex	Gad1+, Gad2+	yes (EX,EC,AS,OPC,OL)	seizure (PTZ)	snRNA-Seq	4
1700016P 03Rik	cortex	Gad1+, Gad2+	yes (EX,EC,MG, OPC, OL)	seizure (PTZ)	snRNA-Seq	4
Nr4al	Vis. CX	all IN	yes (EX,OPC,EC,SM,MA,AS)	visual stim.	scRNA-seq	5
Nr4a2	Vis. CX	SOM+, VIP+,Npy+	yes (EX,EC,MA,AS)	visual stim.	scRNA-seq	5
Efhd2	HPC	VIP+*	yes in EX_DG	NE	snRNA-Seq	6
Baiap2	HPC	VIP+*	yes in EX_DG	NE	snRNA-Seq	6
Gml5425	HPC	VIP+*	yes in EX_DG	NE	snRNA-Seq	6
Bcas2	HPC	VIP+*	yes in EX_DG	NE	snRNA-Seq	6
Gm25603	HPC	VIP+*	not in EX (DG or CAl)	NE	snRNA-Seq	6
Hdac9	HPC	VIP+*	not in EX (DG or CAl)	NE	snRNA-Seq	6
Idh3b	HPC	VIP+*	not in EX (DG or CAl)	NE	snRNA-Seq	6

**TABLE IB**						

**LRG name**	**Brain area**	**IN subtype**	**Activity-dependent induction in other cell types**	**Induction protocol**	**Method**	**Ref.**

Fos2	Vis. CX	Gad2+	yes (EX_Emxl+)	visual stim.	Ribo Tag/Gad2-CRE	1
Gpr3	Vis. CX	Gad2+	yes (EX_Emxl+)	visual stim.	Ribo Tag/Gad2-CRE	1
Rgs2	Vis. CX	Gad2+	yes (EX_Emxl+)	visual stim.	Ribo Tag/Gad2-CRE	1
Vgf	Vis. CX	Gad2+	yes (EX_Emxl+)	visual stim.	Ribo Tag/Gad2-CRE	1
Nptx2	Vis. CX	SOM+	yes (EX_Emxl+)	visual stim.	Ribo Tag/SOM-CRE	1
Gpr3	Vis. CX	SOM+	yes (EX_Emxl+)	visual stim.	Ribo Tag/SOM-CRE	1
E53001K1 ORik	Vis. CX	Gad2+	not in EX_Emxl+	visual stim.	Ribo Tag/Gad2-CRE	1
Pnoc	Vis. CX	Gad2+	not in EX_Emxl+	visual stim.	Ribo Tag/Gad2-CRE	1
Pthlh	Vis. CX	Gad2+	not in EX_Emxl+	visual stim.	Ribo Tag/Gad2-CRE	1
Pdlim3	Vis. CX	PV+	not in EX_Emxl+	visual stim.	Ribo Tag/PV-CRE	2
Prss23	Vis. CX	PV+	not in EX_Emxl+	visual stim.	Ribo Tag/PV-CRE	2
Crhbp	Vis. CX	SOM+, PV+	no other cell type	visual stim.	scRNA-seq	5
Nefm	Vis. CX	only in SOM+	no other cell type	visual stim.	scRNA-seq	5
Frmpd3	Vis. CX	SOM+	not in EX_Emxl+	visual stim.	Ribo Tag/SOM-CRE	1
Slc25a36	Vis. CX	SOM+	not in EX_Emxl+	visual stim.	Ribo Tag/SOM-CRE	1
Prdml	Vis. CX	SOM+	not in EX_Emxl+	visual stim.	Ribo Tag/SOM-CRE	2
Rasllla	Vis. CX	SOM+	not in EX_Emxl+	visual stim.	Ribo Tag/SOM-CRE	2
Serpinel	Vis. CX	SOM+	not in EX_Emxl+	visual stim.	Ribo Tag/SOM-CRE	2
Crh	Vis. CX	VIP+,SOM+, PV+	not in EX_Emxl+	visual stim.	Ribo Tag/VIP-CRE	2
	Vis. CX	VIP+, SOM+, CCK+	no other cell type	visual stim.	scRNA-seq	5
Igfl	Vis. CX	Gad2+	not in EX_Emxl+	visual stim.	Ribo Tag/Gad2-CRE	1
	Vis. CX	VIP+	not in EX_Emxl+	visual stim.	Ribo Tag/VIP-CRE	2
	Vis. CX	only in VIP+	no other cell type	visual stim.	scRNA-seq	5
Igfbp5	Vis. CX	only in VIP+	no other cell type	visual stim.	scRNA-seq	5
Serpine2	Vis. CX	only in VIP+	no other cell type	visual stim.	scRNA-seq	5
Gpdl	Vis. CX	VIP+	not in EX_Emxl+	visual stim.	Ribo Tag/VIP-CRE	2
Hnflb	Vis. CX	VIP+	not in EX_Emxl+	visual stim.	Ribo Tag/VIP-CRE	2
Lpar2	Vis. CX	VIP+	not in EX_Emxl+	visual stim.	Ribo Tag/VIP-CRE	2
Pde3a	Vis. CX	VIP+	not in EX_Emxl+	visual stim.	Ribo Tag/VIP-CRE	2
Prok2	Vis. CX	VIP+	not in EX_Emxl+	visual stim.	Ribo Tag/VIP-CRE	2
Scgn	Vis. CX	VIP+	not in EX_Emxl+	visual stim.	Ribo Tag/VIP-CRE	2
Uts2r	Vis. CX	VIP+	not in EX_Emxl+	visual stim.	Ribo Tag/VIP-CRE	2
Aqp5	Vis. CX	VIP+	not in EX_Emxl+	visual stim.	Ribo Tag/VIP-CRE	2

*A list of activity-dependent genes induced in inhibitory neurons (IN) was selected among hundreds of IEGs and LRGs profiled in the studies reviewed herein. The list is subdivided between IEGs (Table 1A; upregulated at 1 h from induction) and LRGs (Table 1B; upregulated at 4-6 h from induction). The brain areas analyzed are indicated as follows: visual cortex (Vis.CX); medial amygdala (meA); cortex; Hippocampus (HPC). Where possible, induction in specific inhibitory neuron subtypes (classified based on inhibitory markers expression) is indicated for each activity-dependent gene and for each study (IN subtype column). Activity-dependent induction of each gene in cell types other than inhibitory neurons is also reported. Cell types are indicated as follows: excitatory neurons (EX); excitatory neurons expressing Emx1 (EX_Emx1+); Dentate Granule cells (EX_DG); CA1 pyramidal cells (EX_CA1); astrocytes (AS); endothelial cells (EC); oligodendrocytes (OL); oligodendrocyte precursor cells (OPC); microglia (MG); macrophages (MA); smooth muscle cells (SM); pericytes (PE); mural cells (MC; composed of PE and SM). The induction protocols are indicated in the corresponding column as follows: Visual stimulation (visual stim.); seizure upon PTZ injection [seizure (PTZ)]; novel environment exploration (NE). The methods utilized to detect IEG and LRG expression levels are indicated in the corresponding column. References (Ref.) are indicated as follows: Spiegel et al., 2014 (1); Mardinly et al., 2016 (2); Wu et al., 2017 (3); Hu et al., 2017 (4); Hrvatin et al., 2018 (5); Jaeger et al., 2018 (6). Genes specifically induced upon remote memory recall in inhibitory neurons (Chen et al., 2020) are not reported. The asterisk indicates that VIP+ neurons were the only inhibitory subtype profiled in the study.*

### Activity-Dependent Transcription Studies With Single-Cell Resolution

These pioneer studies radically advanced our understanding of activity-dependent transcriptional programs in inhibitory and excitatory neurons and started deciphering the molecular mechanisms that link experience-dependent transcriptional responses to the long-lasting synaptic adaptations of different neuronal subtypes. However, the availability of only a few subtype-specific genetic tools, such as the mouse CRE lines, limited the depth and breadth of these analyses, not capturing the full range of cell-types that compose the nervous system and that respond to activity. Only the advent of RNA sequencing from single-nuclei (snRNA-seq) or single-cells (scRNA Seq) could allow such a comprehensive analysis, and several recent studies have undertaken this important task with significant results. Pioneering this field, Lacar et al. (2016) investigated the transcriptional response of hippocampal dentate granule cells (DGC) at a single-neuron level in mice exposed to a novel environment. When first adopting common whole-cell dissociation protocols, the authors found that the conventional protease digestion step was alone able to induce IEG expression unspecifically and to a degree similar that observed upon seizure. To overcome this hurdle, they resorted to dounce-homogenization of the tissue and FACS isolation of nuclei upon immunostaining for neuronal and DGC markers. Subsequent transcriptome profiling of these nuclei and sorting based on FOS expression, revealed that this approach allows for the detection of IEG expression in behaviorally activated DGC neurons, largely confirming the induction of known IEGs (Arc, Fos, and Erg1) and of their downstream targets. A similar approach was also utilized in a subsequent study by Hu et al. (2017) to resolve the transcriptional heterogeneity of cortical neuronal and non-neuronal cells. In this case, the authors combined the Drop-Seq microfluidic technique (Macosko et al., 2015) with single-nucleus RNA Seq. Also, nuclei from mouse cortices were sucrose-gradient purified rather than isolated by flow cytometry. When applying this novel “sNucDrop-Seq” method to interrogate the transcriptional response to Pentylenetetrazole (PTZ)-induced seizures, the authors find that among the inhibitory neuronal clusters identified, the two subtypes more likely to express activity-dependent genes are the SOM+ and Ndnf+ interneurons. Conversely, and in agreement with previous and subsequent reports (Sørensen et al., 2016; Hrvatin et al., 2018), PV+ interneurons are relatively less transcriptionally responsive to stimulation. Characterization of nuclear rather than whole-cell RNAs allows to better capture the dynamic and transitory expression profiles of activity-dependent genes, as it is not tainted by exported mRNAs which may endure for hours in the cytoplasm. This advantage provided for an additional important finding of this study regarding the timing of activity-dependent transcription in inhibitory neurons. In particular, Hu et al. observed that at 1 h from PTZ treatment the nuclei of inhibitory and excitatory neurons were differentially enriched of IEG and LRG transcripts, with GABAergic cells expressing IEGs (i.e., Fos and Erg1) while principal neurons were already engaged in transcribing LRGs such as Bdnf, Mbnl2, and Nptx2. The observed delay in the transcriptional response of inhibitory neurons suggests that their activation may be triggered subsequently to that of excitatory neurons, in accordance with a current model of inhibitory engram formation as a homeostatic response to the activation of excitatory memory engrams (Barron et al., 2017).

To obtain whole-cell transcriptomic profiles, Wu et al. resolved the issue of unspecific IEG expression induced by conventional dissociation methods with a different strategy (Wu et al., 2017). They observed that such spurious expression was minimized when the protease digestion step was performed at lower temperatures and in the presence of the transcriptional inhibitor actinomycin. They applied this protocol to analyze, at a single-cell level, the transcriptional profiles of cells composing the medial amygdala (MeA), in control animals and in animals undergoing either PTZ-induced seizures or behavioral stimuli like acute stress. Strikingly, they found that activity-induced IEG expression is not a prerogative of neurons and that partially overlapping sets of IEGs can be also detected in many non-neuronal cells, including astrocytes, microglia, oligodendrocytes, endothelial cells and mural cells. When focusing on neuronal subtypes, the authors found that a common set of four IEGs, Fos, Fosb, Egr4, and Nr4a3 (Table 1), was induced by seizure across all MeA neuronal subclusters, partially confirming previous data from Spiegel et al. (2014). However, most of the IEGs characterized displayed differential induction levels in the different clusters. Additionally, the authors were able to identify two neuronal subclusters, both expressing CCK, which were preferentially enriched of IEGs in response to acute stress. Shortly after, a similar study was published investigating the transcriptional response to sensory stimulation of the visual cortex at a single-cell level (Hrvatin et al., 2018). As in the MeA, also in the visual cortex a large array of non-neuronal cells displays strong transcriptional responses to stimulation, particularly noticeable in endothelial and smooth muscle cells, astrocytes, pericytes and macrophages. Further, the authors (Hrvatin et al., 2018) identify 611 genes significantly regulated by sensory stimulation in the various cell types, divided into 362 early-response and 249 late-response genes. When examining their expression levels across the 30 different subtype clusters, they observed great heterogeneity in their distribution, highlighting divergent transcriptional responses both at early and late time points from light stimulation. Interestingly, among 38 IEG transcription factors identified, only half are expressed by three or more cell types, meaning that a large portion of IEGs is expressed only in defined clusters. Of the “shared” IEG transcription factor, a small subset (including Fos, Fosl2, Erg1, Nr4a1-3) are induced in both neuronal and non-neuronal cell types, while five (Npas4, Junb, Fosb, Erg2 and Erg4) are induced only in neurons, particularly in all excitatory sub-clusters and in the SOM+ and VIP+ inhibitory sub-clusters (Table 1). Hence, a more in depth single-cell analysis of experience-regulated gene expression profiles depicts a picture that partially diverges from the initial conclusions drawn by Spiegel et al. (2014). Indeed, if on one hand the existence of a small core of common early transcription factors is confirmed, on the other, the bulk of early transcriptional responses appears to be highly diversified across cell-types and could contribute to drive differential downstream LRG programs. When examining sensory-induced LRG programs, both excitatory and inhibitory sub-clusters display highly divergent transcriptional profiles. In particular, the authors (Hrvatin et al., 2018) were able to identify 14 late-response genes differentially enriched in specific inhibitory subtypes; for example, the LRGs Igf1, Crh, Igfbp5 and Serpin2 are specifically enriched in VIP+ neurons (Table 1). While the role of Igf1 had been previously examined (Mardinly et al., 2016), in this study the authors point to Crh (the Corticotropin-releasing hormone) as a molecule secreted by VIP+ neurons which can increase the excitability of nearby pyramidal cells in response to stimulation (Figure 1). Conversely, SOM+ interneurons secrete an inhibitor of Crh, suggesting a novel mechanism by which sensory-experience shapes neuronal excitability through the opposing effects of LRG-encoded molecules secreted by two different inhibitory subtypes (Figure 1).

Alternative approaches to scRNA-seq, that resolve not only the identity but also the spatial distribution of activity-dependent transcripts, are also emerging (Lein et al., 2017). These include the 3D intact-tissue RNA sequencing “STARmap” methodology, developed by combining hydrogel-tissue chemistry, targeted signal amplification and *in situ* sequencing (Wang et al., 2018). With this approach, Wang et al. (2018) were able to obtain a 3D map single-cell transcriptomic profiles and spatial organization of molecularly defined cell types. This promising technique was also applied to assess the expression pattern of 48 activity dependent genes in the visual cortex at 1 h of visual stimulation. The results were highly consistent with previous scRNA-Seq data, reaffirming the existence of a small set of common IEGs (including Fos and Egr1) and a significant divergence in the expression of all other IEG tested across cell-types. Further, the authors could observe a more pronounced diversification of IEG transcriptional profiles across inhibitory neuron subtypes than across excitatory neuron subtypes, again confirming the conclusions drawn by scRNA-Seq studies.

Finally, a recently published study investigated how activity-dependent transcription, triggered by exploration of a novel environment, drives circuit reorganization by modulating perisomatic inhibitory plasticity onto CA1 pyramidal hippocampal neurons (Yap et al., 2021). The authors focus on the Fos-dependent transcriptional cascade in these cells to characterize LRGs that are responsible for the observed increase in inhibition by PV-interneurons and weakening of CCK-interneurons inputs. To this end, they combine the results of three different profiling approaches; a Ribo-tag analysis of activity-dependent genes in CamkII-positive cells, a snRNA-seq of Fos-depleted nuclei upon kainic acid stimulation and finally, a chromatin-profiling strategy to identify Fos-bound target genes. Six candidate effector LRGs, induced by Fos in CA1 excitatory neurons, stand out from these analyses: Inhba, Bdnf, Scg2, Rgs2, Nptx2 and Pcsk1. Of these, the pro-neuropeptide Scg2 emerged as the Fos-dependent LRG key to modulating perisomatic inhibition by PV and CCK interneurons and able to affect network rhythms. Thus, in CA1 pyramidal neurons, activity-dependent expression of Fos leads to selective induction of Scg2, a precursor to four neuropeptides, which in turn rearranges PV and CCK inhibitory inputs (Figure 1), likely facilitating memory consolidation. This study also revealed that, as observed for Npas4 (Spiegel et al., 2014), Fos-dependent LRG programs differ substantially in the various cell-types analyzed. Consequently, it would be interesting to learn whether Fos-dependent LRGs specifically induced in inhibitory neurons intervene to modulate synaptic plasticity, and possibly network oscillations, similarly to what observed in pyramidal neurons via the LRG Scg2.

### Transcriptional Programs in Memory Recall and Consolidation

The studies presented above all focused on characterizing the transcriptional programs of activated cells within the first few hours from the inducing stimuli, which ranged from pharmacological induction to visual stimulation and novel environment exploration (Table 1). The transcriptional programs characterized are therefore potentially useful to better understand, and possibly manipulate, the acquisition phase of memory encoding or learning. Hence, the question arises as to what happens in the subsequent phases of memory formation and recall. Is activity-dependent transcription modulated during the process of engram selection, consolidation and reactivation, and what is its contribution to each specific stage? Two recent studies started addressing these questions. Adopting the snRNA-seq technique (Lacar et al., 2016; Jaeger et al., 2018) characterized the transcriptional profile of specific subsets of Fos+ hippocampal neurons (CA1 pyramidal neurons, VIP+ interneurons and DGC) at early and late (1 and 5 h) time points from novel environment exposure and upon re-exposition to the same, or a different, environment (after 4 h from the initial exposure). Among several important findings of this study, the authors were able to define an early activity-induced transcriptional signature of DGC neurons that is predictive of their reactivation in the recall phase of the behavioral paradigm; what could be seen as a transcriptional mark for engram cell selection. Further, they find that a significant portion of the transcriptional response of activated DG neurons is devoted to reducing excitability and increasing tonic inhibition of these cells. This evidence suggests an intriguing mechanism of engram ensemble selection in the DG. Activation of the initial network of DG neurons triggers heterogeneous transcriptional programs which also dampen excitability of the activated neurons: only those neurons expressing specific gene patterns can overcome such heightened inhibition and are selected as engram cells encoding that memory. With regard to inhibitory neurons, the authors report that hippocampal Fos+ VIP+ interneurons are transcriptionally less responsive to novel environment exposure compared to CA1 glutamatergic neurons and to DGC. Only seven experience-dependent IEGs appear induced in Fos+ VIP+ neurons (Table 1A), while ten are down-regulated (Jaeger et al., 2018). This result diverges from what observed in the visual cortex upon sensory stimulation (Mardinly et al., 2016; Hrvatin et al., 2018) possibly reflecting differences in the transcriptional responsiveness of cortical versus hippocampal VIP interneurons. Alternatively, selection of activated neurons based on Fos expression may exclude neurons which do not express Fos but are nonetheless transcriptionally active (Sun et al., 2020). Unfortunately, additional studies that provide a comprehensive single-cell analysis of the transcriptional responses in inhibitory neurons upon memory formation are lacking. Conversely, studies that specifically address memory-induced transcriptional programs in activated excitatory neurons are starting to emerge (Rao-Ruiz et al., 2019; Yap et al., 2021).

However, a recent study was published which directly investigated the transcriptional profiles of cortical engram cells activated 16 days post-training, in a fear memory recall paradigm (Chen et al., 2020). This first profiling of consolidated engram cell composition, and of gene expression patterns associated with remote memory recall, brought important findings to light. First, that the relative composition in neuronal subtypes of active and inactive neurons is highly similar, albeit for an enhanced recruitment of Calb2+ GABAergic interneurons within the reactivated ensemble. Secondly, that cell type-specific persistent transcriptional programs are elicited during memory consolidation in both neuronal and non-neuronal engram cells, such as astrocytes and microglia. These gene expression profiles are long lasting and likely contribute to maintaining the memory trace at remote time points after learning. Intriguingly, the authors also find that within these memory-related transcriptional programs, a large portion of activated genes are related to vesicle-mediated transport, exocytosis and neurotransmitter secretion. This implies a strong participation of these cellular processes in memory consolidation.

## Conclusion and Perspectives

Overall, in recent years high-throughput approaches have significantly accelerated the discovery of stimulus-dependent transcriptional profiles across a surprisingly vast array of neuronal and non-neuronal cell-types. It needs to be noted that, with a few recent exceptions, most of these investigations interrogated the transcriptional response of heterogenous brain areas to pharmacological or sensory stimulation, not to memory-inducing protocols. This is particularly true with respect to GABAergic neurons (Table 1). To our knowledge, only one single-cell transcriptomic study profiled the transcriptional program of inhibitory neurons upon memory formation (Jaeger et al., 2018), limiting the analysis to the hippocampal Fos+ VIP+ cluster. Indeed, the study by Chen et al. (2020) specifically investigated the transcriptional signature of both inhibitory and excitatory neurons upon remote memory recall. Moreover, the lack of uniformity in experimental approaches, while enriching, complicates an already intricate picture of sub-type specific transcriptional responses to activity. Hence, it is premature to ascribe specific transcriptional responses to different neuronal subclasses upon memory formation. Nonetheless, important hints can be extrapolated from these pioneer studies, albeit requiring further validation in memory-related processes. Except for a small set of immediate-early genes expressed in multiple cell-types, both the early and late transcriptional responses appear to be markedly subtype-specific. This feature is particularly noticeable across the different classes of interneurons, likely reflecting a higher degree of functional diversification among GABAergic subtypes. Further, this diversity will surely be instrumental in the development of genetic tools to map and manipulate the activation of specific subtypes of neuronal and non-neuronal cells.

The significance of these studies, however, goes well beyond the potential advancement in memory trace labeling techniques. As described above, characterization of cell type-specific transcriptional responses to activity has led to the unveiling of complex and varied molecular cascades that underlie stimulation-dependent circuit reorganization. This is best exemplified by the opposite effects on synaptic connectivity triggered by Npas4 in excitatory versus inhibitory neurons (Figure 1). Similarly, Fos appears to induce highly diversified LRG transcriptional programs specific to each cellular subtype, likely mediating different rearrangements of their synaptic connections within their neuronal network. Additional complexity stems from heterogeneous IEG expression, as suggested by the identification of distinct engram ensembles in the dentate gyrus whose function and circuit reorganization are differentially directed by Fos and Npas4 transcriptional programs (Sun et al., 2020).

A common theme that emerges from these investigations, is that activity-dependent transcriptional programs are elicited and persist in both neuronal and non-neuronal cells, particularly in astrocytes and microglia. Thus, memory engrams likely consist of a heterogeneous network of cells, including excitatory neurons, inhibitory neurons and, intriguingly, glia cells. In support of this, several functional studies point to microglia, astrocytes and oligodendrocytes, as fundamental cellular subtypes driving and sustaining plasticity and memory (Parkhurst et al., 2013; Adamsky et al., 2018; Nguyen et al., 2020; Pan et al., 2020; Steadman et al., 2020; Zhou et al., 2021).

Another aspect to which several studies converge, is that activity-dependent transcriptional responses, in both excitatory and inhibitory neurons, are often directed at increasing the number or strength of inhibitory inputs onto activated pyramidal neurons (Bloodgood et al., 2013; Spiegel et al., 2014; Mardinly et al., 2016; Hrvatin et al., 2018; Jaeger et al., 2018; Sun et al., 2020; Yap et al., 2021). Consequently, the question arises: how does an increased inhibitory drive onto the activated excitatory ensemble contribute to engram formation? A possible answer is that this homeostatic response may represent a mechanism for the allocation of the inhibitory component of the engram and for shaping engram size, whereby inhibitory engram neurons are recruited by, and in response to, activation of excitatory ensemble neurons. This hypothesis would be also in accordance with a model of “inhibitory engram” formation proposed by Barron et al. (2017) and with the observation of a delayed transcriptional response to activity in inhibitory neurons (Hu et al., 2017).

Nonetheless, an increased inhibitory tone onto activated pyramidal cells needs to be reconciled with consolidated evidence showing that heightened intrinsic excitability of activated ensemble neurons is critical for their allocation to the engram (reviewed in Yiu et al., 2014; Josselyn and Frankland, 2018; Josselyn and Tonegawa, 2020). Thus, how can heightened excitability and increased inhibitory tone coexist in activated ensemble pyramidal neurons? And how do they functionally cooperate to the process of engram allocation? As suggested above (Morrison et al., 2016; Stefanelli et al., 2016; Jaeger et al., 2018), heightened inhibition of activated glutamatergic neurons may contribute to restrict engram allocation to a discrete subset of activated neurons, likely those with an increased intrinsic excitability. In this light, we propose two possible solutions to this apparent paradox.

**FIGURE 2:**
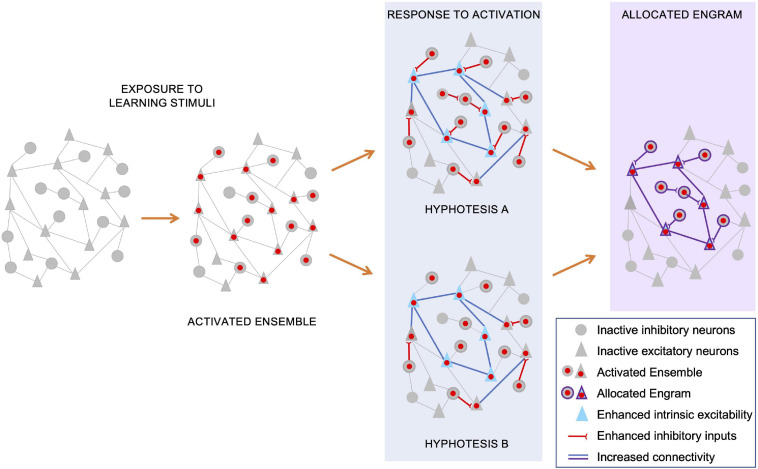
Potential mechanisms underlying allocation of the inhibitory and excitatory components of the engram. During memory acquisition (“exposure to learning stimuli”), an initial ensemble of inhibitory and excitatory neurons are activated, and their nuclei (red dots) undergo activity-dependent IEG/LRG transcription. The consequent expression of activity-dependent genes, combined with synaptic plasticity changes triggered by neuronal activation, leads to circuit-wide rearrangements of neuronal connectivity. For a subset of pyramidal neurons, these rearrangements include both enhanced intrinsic excitability (blue triangles) and increased synaptic strength among their synapses (blue lines). Concomitantly, activation of both inhibitory and excitatory neurons elicits an enhancement in strength and number of inhibitory inputs onto activated pyramidal neurons. Independent reports suggest that both heightened inhibition onto excitatory neurons and their increased intrinsic excitability contribute to restrict engram allocation. Regarding a potential underlying mechanism, we suggest two, non-exhaustive, scenarios. In a first scenario (hypothesis A), increased inhibitory inputs could be induced onto most, if not all, activated pyramidal neurons. Consequently, only those neurons with an intrinsic excitability able to override this increased inhibitory drive can win the competition for engram allocation. In this scenario, those inhibitory neurons targeting successfully allocated engram cells could become the inhibitory component of that engram, controlling its storage and recall. In an alternative hypothesis (hypothesis B), heightened inhibition and increased intrinsic excitability do not co-exist, targeting distinct subsets of activated pyramidal neurons. Those ensemble pyramidal neurons that receive increased inhibitory inputs would be excluded from allocation, while those with an increased intrinsic excitability would be more likely selected as engram neurons. In this model, those GABAergic neurons initially recruited to restrict the allocation process, would most likely be excluded from the consolidated engram ensemble. Hence, the inhibitory component of the allocated engram may be recruited in a subsequent phase, possibly as a homeostatic response to the increased excitation of engram pyramidal neurons.

In a first scenario (Figure 2, hypothesis A), an increase in inhibitory inputs could be a generalized response to the initial stimuli, targeting pyramidal neurons of the activated ensemble. Only those neurons with a heightened intrinsic excitability would be able to override this increased inhibitory drive and win the competition for engram allocation. In this scenario, those inhibitory neurons targeting successfully allocated engram cells could become the inhibitory component of that engram, controlling its storage, latency and recall.

In an alternative hypothesis (Figure 2, hypothesis B), excitability and inhibition may act on distinct subsets of activated pyramidal neurons to restrict engram size. Those ensemble pyramidal neurons that receive increased inhibitory inputs would be largely excluded from allocation, while those with an increased intrinsic excitability would be more likely selected as engram neurons. In this scenario, those inhibitory neurons initially recruited to restrict the allocation process, would most likely be excluded from the consolidated engram ensemble. In this case, the inhibitory component of the allocated engram may be recruited in a homeostatic response to the increased excitation of engram pyramidal neurons.

These proposed allocation mechanisms are clearly non-exhaustive. Nonetheless, to test them it would be useful to ascertain whether pyramidal neurons that are activated during memory acquisition, and that display increased intrinsic excitability, also receive increased inhibitory inputs.

Numerous other aspects regarding the recruitment and activity of the inhibitory component of memory engrams remain unclear. For instance, it would be useful to determine if those inhibitory neurons that are activated in the first phase of engram allocation are the same inhibitory cells that control storage and recall of that memory trace. Further investigations are also needed to establish the role played by disynaptic disinhibition in these processes. It is conceivable that disinhibitory mechanisms may play a key role in memory recall, relieving inhibition and allowing reactivation of excitatory engram cells. Similarly, disinhibition may contribute to engram allocation by enhancing excitation of pyramidal engram cells. However, compelling experimental evidence that support these hypotheses is still lacking. The rapid expansion of studies on the role of inhibitory neurons in memory processes, and the constant development of new genetic tools that can access engram cells with subtype specificity, will undoubtedly provide clues to address these challenging questions. Finally, transcriptomic analyses have unveiled experience-dependent widespread activation of non-neuronal cells, including glia, endothelial cells and macrophages. Understanding how the activity of these cells may contribute to modulate both excitatory and inhibitory engram neurons is surely a new and intriguing area to investigate in memory engram studies.

## Author Contributions

Both authors conceived and wrote the manuscript and approved the submitted version.

## Conflict of Interest

The authors declare that the research was conducted in the absence of any commercial or financial relationships that could be construed as a potential conflict of interest.
